# Draft Genome Sequence of the Gammaproteobacterial Strain MOLA455, a Representative of a Ubiquitous Proteorhodopsin-Producing Group in the Ocean

**DOI:** 10.1128/genomeA.01203-13

**Published:** 2014-01-30

**Authors:** Alicia Courties, Thomas Riedel, Michael Jarek, Maria Papadatou, Laurent Intertaglia, Philippe Lebaron, Marcelino T. Suzuki

**Affiliations:** a Sorbonne Universités, UPMC Univ Paris 06, USR 3579, LBBM, UMS2348 (Plate-forme Bio2Mar) Observatoire Océanologique, Banyuls/Mer, France; b CNRS, USR 3579, LBBM, Observatoire Océanologique, Banyuls/Mer, France; c Helmholtz-Centre for Infection Research, Genome Analytics, Braunschweig, Germany; d CNRS, UMS 2348 (Plate-forme Bio2Mar) Observatoire Océanologique, Banyuls/Mer, France

## Abstract

Strain MOLA455 is a marine gammaproteobacterium isolated from the bay of Banyuls-sur-Mer, France. Here, we present its genome sequence and annotation. Genome analysis revealed the presence of genes associated with a possibly photoheterotrophic lifestyle that uses a proteorhodopsin protein.

## GENOME ANNOUNCEMENT

Strain MOLA455 was isolated from a coastal water sample taken from a depth of 3 m at station SOMLIT Observatory Laboratoire Arago (SOLA), located 0.5 mi offshore in the bay of Banyuls-sur-Mer, France (42°29.300′N 3°08.700′E). This strain belongs to the ubiquitous gammaproteobacterial SAR92 group. Its 16S rRNA was found to be 99% identical to an operational taxonomic unit (OTU) shown to be very abundant in surface ocean water around the world (1).

For whole-genome sequencing, the strain was cultivated in Marine Broth 2216 medium (BD, Difco, Sparks, MD) at 25°C for 2 weeks. The cetyltrimethylammonium bromide (CTAB) method was used for genomic DNA isolation (2), except that all used volumes were divided by two. The library for whole-genome sequencing was prepared using the TruSeq DNA PCR-free sample preparation kit (Illumina, San Diego, CA), with 550-bp insert sizes, according to the manufacturer’s protocol. The genomic DNA was fragmented using the Covaris S2 system (Covaris, Woburn, MA). Overhangs were converted into blunt ends. Additionally, A-bases were added to the 3′ end of the blunt phosphorylated DNA fragments, followed by purification and multiple indexed adapter ligation. The quality of the prepared library was checked by using quantitative PCR (qPCR) (Kapa library quantification kit, Kapa Biosystems, Woburn, MA), as well as on an Agilent Bioanalyzer high-sensitivity (HS) chip (Agilent Technologies, Santa Clara, CA), performed according to the manufacturer’s instructions. For paired-end genome sequencing, a MiSeq System (Illumina) was used, resulting in 2,640,464 reads, 2,365,483 of which were finally converted to fastq format and *de novo* assembled with Velvet 1.2.07 (3). The fastq-mcf tool of ea-utils (http://code.google.com/p/ea-utils) was used to control the sequencing data for general quality features. The resulting 4 scaffolds of the genome, with 161× average coverage, were annotated using Prokka 1.7 (http://www.vicbioinformatics.com/software.prokka.shtml).

The sequenced draft genome of strain MOLA455 consists of 4 contigs, totaling 2,605,026 bp in size, and it has a G+C content of 50.02%. It was found to encode 2,331 coding sequences, 3 rRNAs, and 35 tRNAs.

Genome analysis revealed the presence of a green-light-absorbing proteorhodopsin-encoding sequence (PR) of 229 amino acid residues (4–6). It shows sequence features suggestive of proton pump activity from the inside to the outside of the bacterial cell, leading to a proton motive force (*pmf*) across the cell membrane (4, 6, 7). In addition, genes associated with a retinal-producing pathway were detected (7–9)

BLAST analysis (10) showed the highest PR protein sequence identity to the PR sequence of the marine gammaproteobacterium HTCC 2207, which was reported to reach up to 10% of the total bacterioplankton in surface waters close to the coast of Oregon (11). The presence of a PR gene sequence, together with gene sequences putatively associated with retinal biosynthesis in the genome sequence of strain MOLA455, suggest a putative photoheterotrophic lifestyle that generates energy from light.

### Nucleotide sequence accession numbers.

The whole-genome shotgun project has been deposited at DDBJ/EMBL/GenBank under the accession no. AZIN00000000. The version described in this paper is version AZIN01000000.
